# Supplementary figures and images for: Reverse causation bias: A simulation study comparing first- and second-line treatments with an overlap of symptoms between treatment indication and studied outcome
Source: PLoS One. 2024 Jul 12;19(7):e0304145. doi: 10.1371/journal.pone.0304145 (PMC11244844; doi:10.1371/journal.pone.0304145)

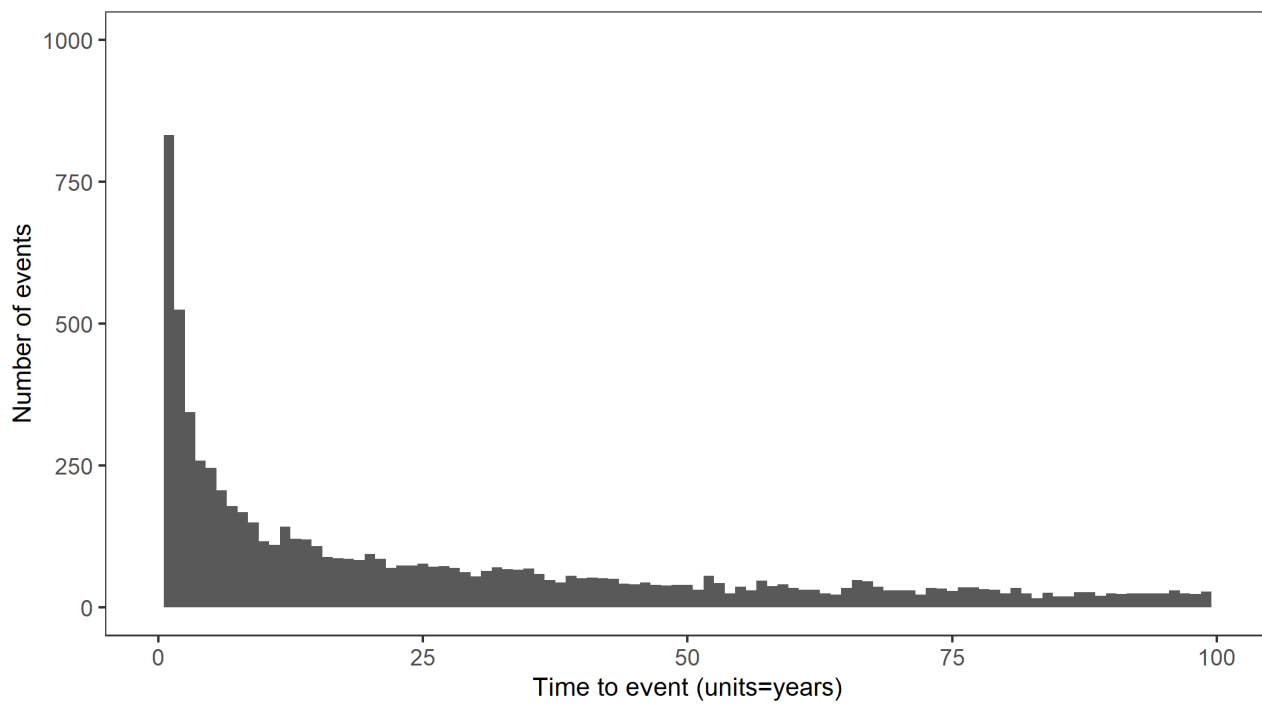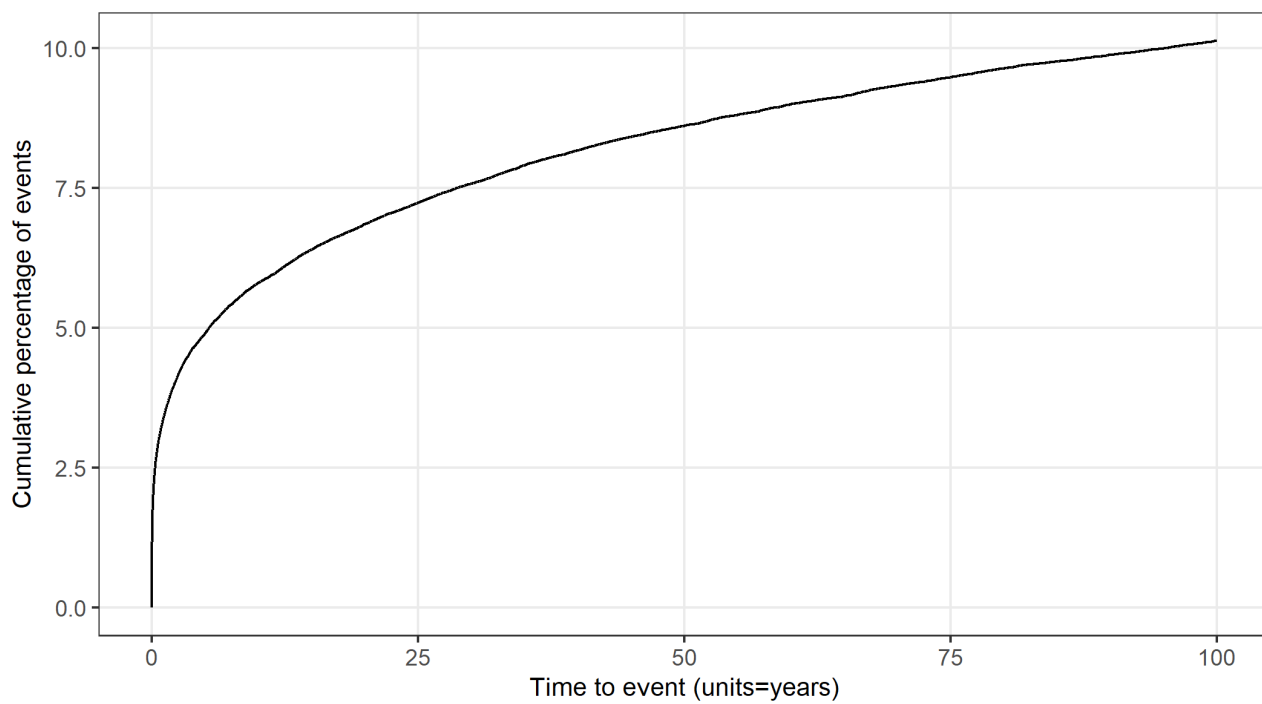

Supplement: S1 Fig — (PDF) [file pone.0304145.s001.pdf]

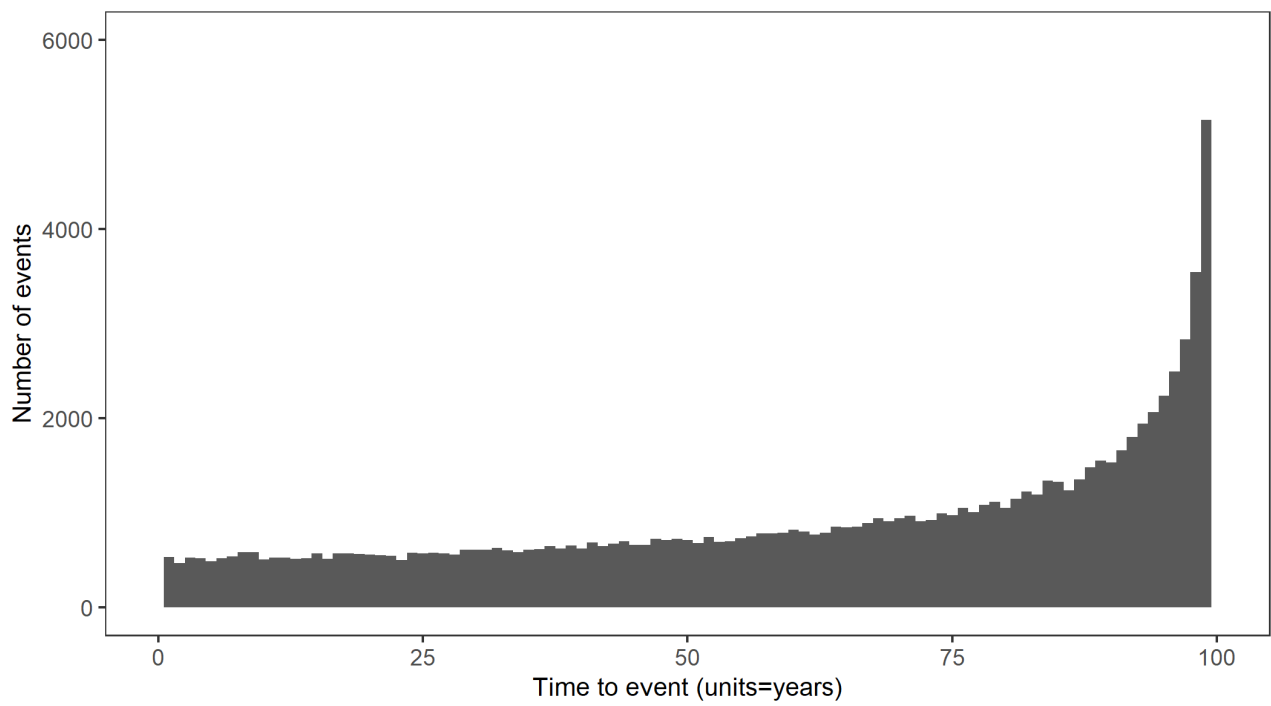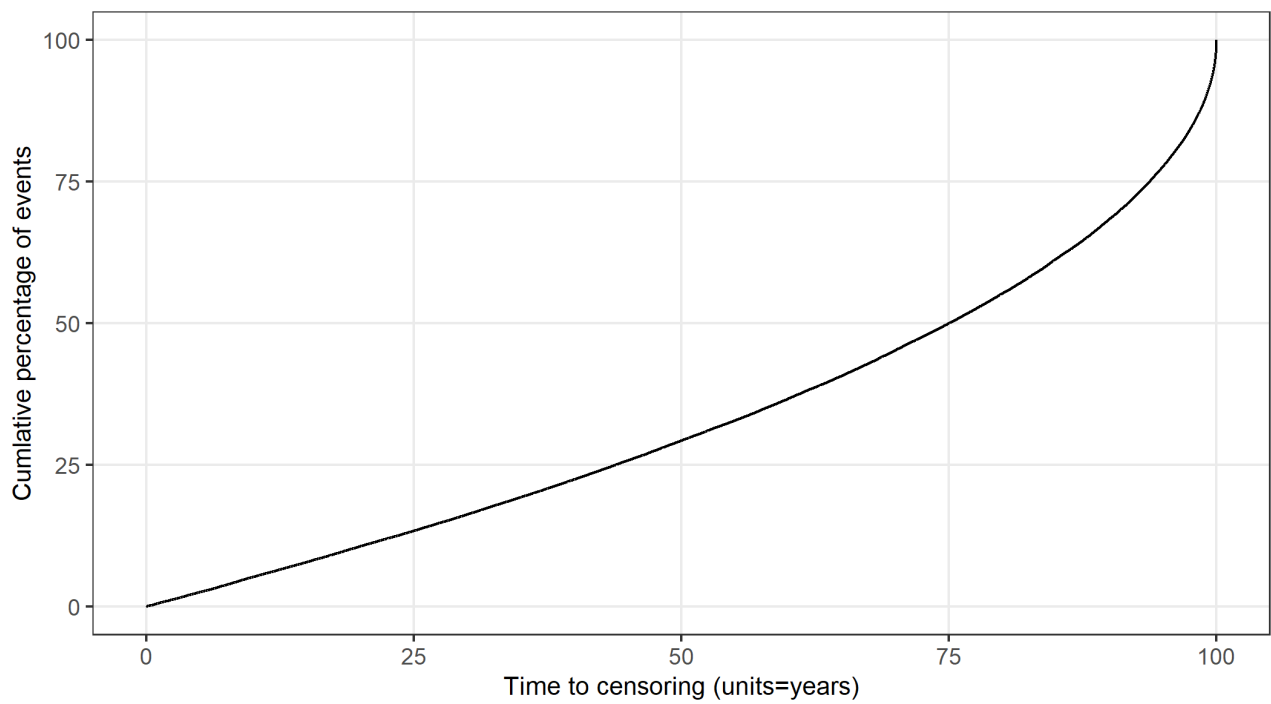

Supplement: S2 Fig — (PDF) [file pone.0304145.s002.pdf]

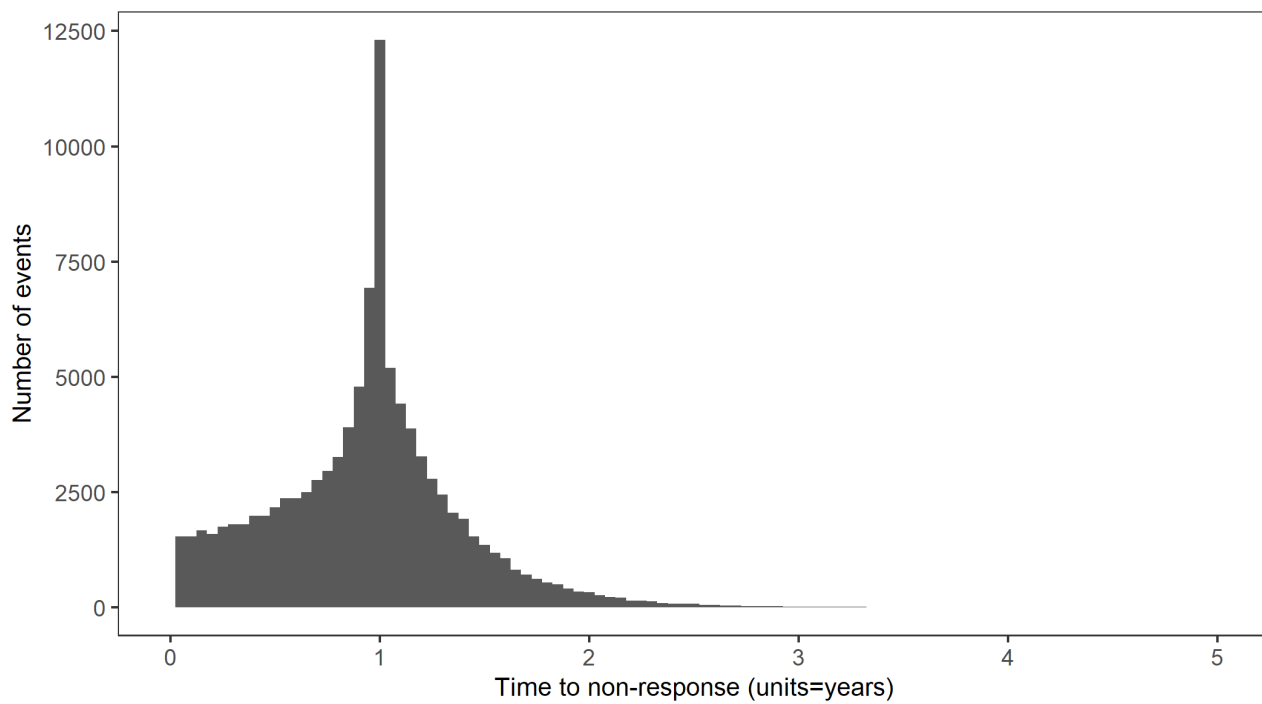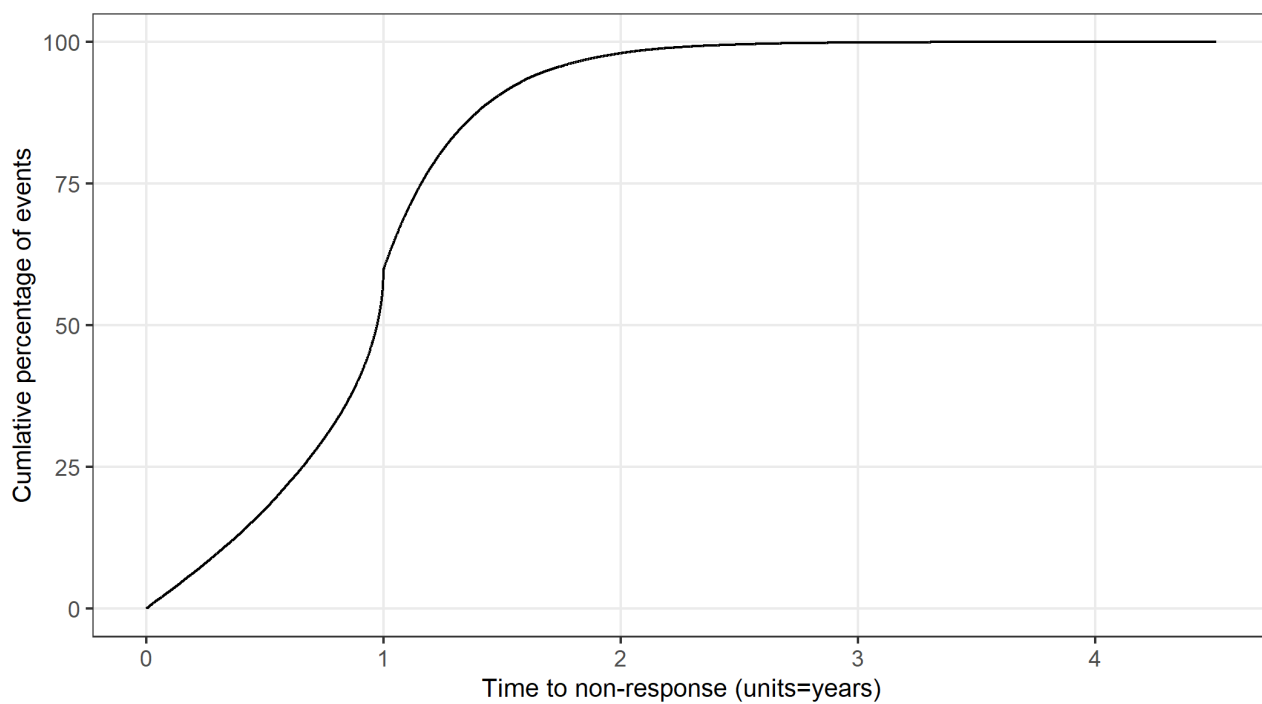

Supplement: S3 Fig — (PDF) [file pone.0304145.s003.pdf]

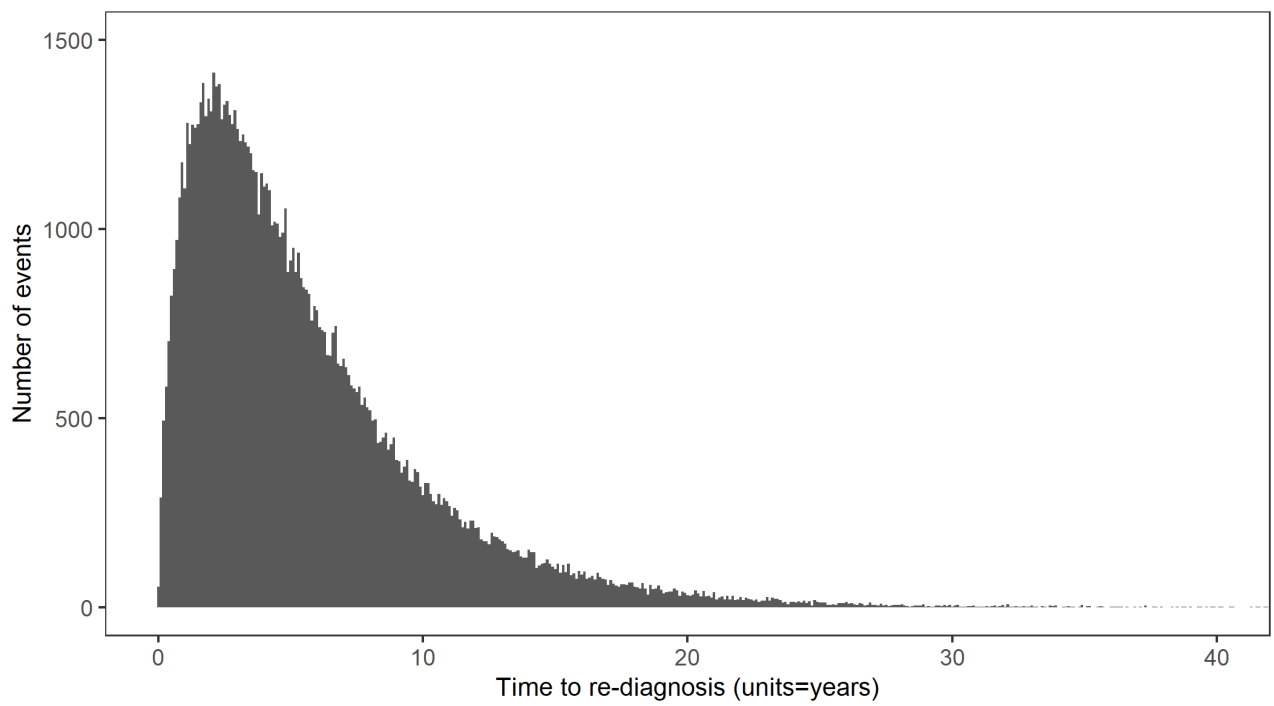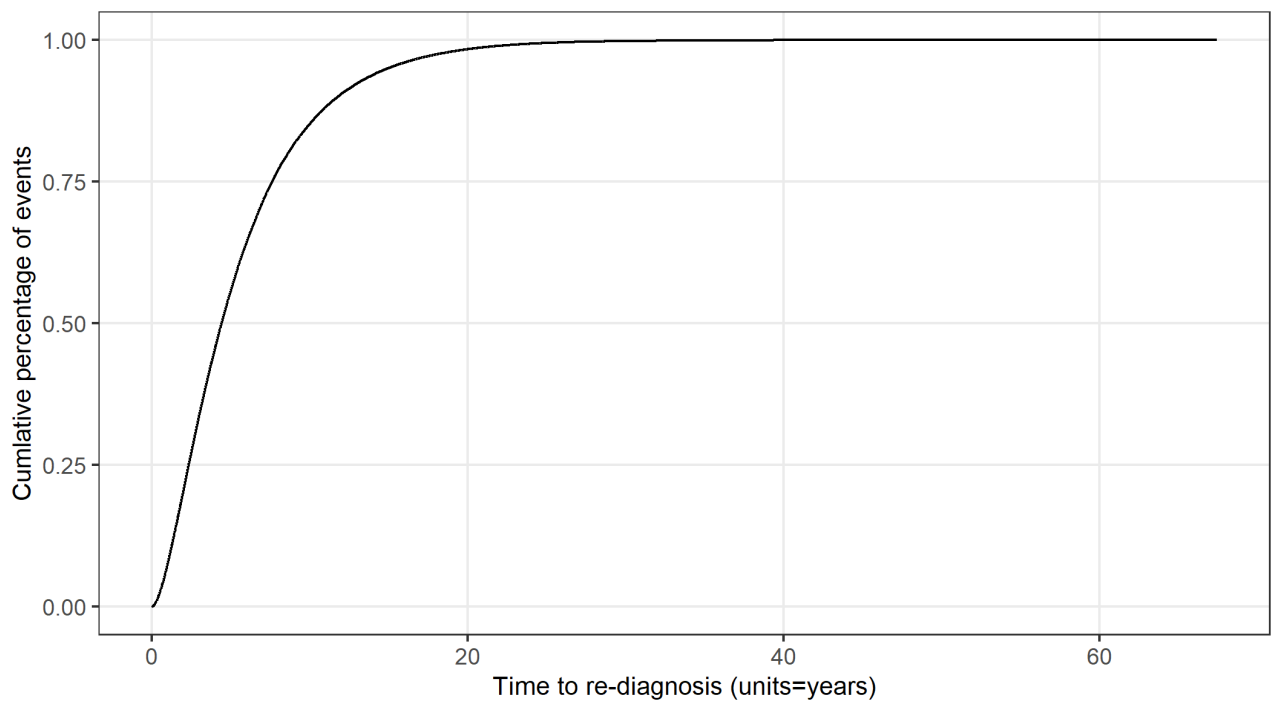

Supplement: S4 Fig — (PDF) [file pone.0304145.s004.pdf]
