# Supplementary material for: Reverse causation bias: A simulation study comparing first- and second-line treatments with an overlap of symptoms between treatment indication and studied outcome
Source: PLoS One. 2024 Jul 12;19(7):e0304145. doi: 10.1371/journal.pone.0304145 (PMC11244844; doi:10.1371/journal.pone.0304145)
Supplement: S1 File — (ZIP) [file pone.0304145.s005.zip › S1_File.pdf]

this is the simulation code
First helper and simulation functions will be defined
Then the simulation set will be defined immediately followed by a for-loop running the simulations

helper functions (functions and packages) in here
mstate functions - had to change a bit of coding in one to enable the transition when only 1 subject is transitioning.

```
{
  transMat <- function(x, names) {
    ## transMat:  produce transition matrix for use in package 'mstate'.
    ## Arguments:
    ## x:  List of possible transitions.
    ##     x[[i]] consists of a vector of state numbers
    ##     reachable from state i.
    ## names: Character vector of state names, having the same length
    ##        as x.
    ## Example:  States 1 and 2 are reachable from one
    ##     another.  State 3 is absorbing, reachable from
    ##     states 1 and 2.
    ##     transMat( x = list( c(2, 3), c(1, 3), c() ) )
    if ( !is.list(x) ) stop("x must be a list")
    ns <- length(x) ## number of states
    tmat <- matrix(NA, nrow = ns, ncol = ns) ## transition matrix
    if ( missing(names) ) {
      if ( !is.null( base::names(x) ) ) {
        namesList <- list(from = base::names(x), to = base::names(x))
      } else {
        namesList <- list(from = paste("State", seq(nrow(tmat))),
                          to = paste("State", seq(nrow(tmat))))
      }
    } else {
      if ( length(names) != ns ) stop("length of 'names' must equal length of 'x'")
      namesList <- list(from = names, to = names)
    }
    idxmat <- cbind(unlist(lapply(seq(ns),
                                  function(i, y){
                                    rep(i, length(y[[i]]))}, x)),
                    unlist(x))
    if ( max(idxmat) > ns )
      stop("Largest state in transition list exceeds number of states")
    tmat[idxmat] <- seq(nrow(idxmat))
    dimnames(tmat) <- namesList
    tmat
  }
```

```
msprep <- function (time, status, data, trans, start, id, keep)
{
 if (!(is.matrix(time) | (is.data.frame(time)))) {
   if (!is.character(time))
     stop("argument \"time\" should be a character vector")
   if (missing(data))
     stop("missing \"data\" argument not allowed when time argument is character vector")
   startings <- which(apply(!is.na(trans), 2, sum) == 0)
   wh <- which(is.na(time))
   if (!all(wh %in% startings))
     stop("no NA's allowed in the \"time\" argument for non-starting states")
   tcols <- match(time[!is.na(time)], names(data))
   if (any(is.na(tcols)))
     stop("at least one of elements of \"time\" not in data")
   time <- matrix(NA, nrow(data), length(time))
   whcols <- (1:ncol(time))[!(1:ncol(time)) %in% wh]
   time[, whcols] <- as.matrix(data[, tcols])
 }
 if (!(is.matrix(status) | (is.data.frame(status)))) {
   if (!is.character(status))
     stop("argument \"status\" should be a character vector")
   if (missing(data))
     stop("missing \"data\" argument not allowed when status argument is character vector")
   startings <- which(apply(!is.na(trans), 2, sum) == 0)
   wh <- which(is.na(status))
   if (!all(wh %in% startings))
     stop("no NA's allowed in the \"status\" argument for non-starting states")
   dcols <- match(status[!is.na(status)], names(data))
   if (any(is.na(dcols)))
     stop("at least one of elements of \"status\" not in data")
   status <- matrix(NA, nrow(data), length(status))
   whcols <- (1:ncol(status))[!(1:ncol(status)) %in% wh]
   status[, whcols] <- as.matrix(data[, dcols])
 }
 time <- as.matrix(time)
 status <- as.matrix(status)
 if (!all(dim(time) == dim(status)))
   stop("unequal dimensions of \"time\" and \"status\" data")
 n <- nrow(time)
 K <- dim(trans)[1]
 if ((dim(trans)[2] != K) | (dim(time)[2] != K))
   stop("dimension of \"trans\" does not match with length of \"time\" and \"status\"")
 idname <- "id"
 missingid <- FALSE
 if (missing(id)) {
   missingid <- TRUE
   id <- 1:n
 }
```

```
else {
  if (!is.vector(id))
    stop("argument \"id\" is not a vector")
  else {
    if (!is.character(id)) {
      if (length(id) != n)
        stop("argument \"id\" not of correct length")
    }
    else {
      if (length(id) == 1) {
        if (n == 1)
          stop("cannot determine whether \"id\" argument indicates ")
        else {
          idname <- id
          id <- data[[id]]
        }
      }
      else {
        if (length(id) != n)
          stop("argument \"id\" not of correct length")
        id <- factor(id)
      }
    }
  }
}
idlevels <- NULL
if (is.factor(id))
  idlevels <- levels(id)
if (!missing(start)) {
  startstate <- start$state
  starttime <- start$time
  if (length(startstate) != length(starttime))
    stop("starting states and times not of equal length")
  if (length(startstate) > 1) {
    if (length(startstate) != n)
      stop("length of starting states and times different from no of subjects in data")
  }
  else {
    startstate <- rep(startstate, n)
    starttime <- rep(starttime, n)
  }
}
else {
  startstate <- rep(1, n)
  starttime <- rep(0, n)
}

ord1 <- order(id)
```

```
msres <- msprepEngine(time = time, status = status, id = id,
                      starttime = starttime, startstate = startstate, trans = trans,
                      originalStates = (1:nrow(trans)), longmat = NULL)
msres <- as.data.frame(msres)
names(msres) <- c(idname, "from", "to", "trans", "Tstart",
                  "Tstop", "status")
msres$time <- msres$Tstop - msres$Tstart
msres <- msres[, c(1:6, 8, 7)]
ord <- order(msres[, 1], msres[, 5], msres[, 2], msres[, 3])
msres <- msres[ord, ]
row.names(msres) <- 1:nrow(msres)
if (!is.null(idlevels))
  msres[, 1] <- factor(msres[, 1], 1:length(idlevels),
                       labels = idlevels)
if (!missing(keep)) {
  if (!(is.matrix(keep) | (is.data.frame(keep)))) {
    if (is.character(keep)) {
      if (missing(data))
        stop("argument \"data\" is missing, with no default")
      nkeep <- length(keep)
      kcols <- match(keep, names(data))
      if (any(is.na(kcols)))
        stop("at least one of elements of \"keep\" not in data")
      keepname <- keep
      keep <- data[, kcols]
    }
    else {
      nkeep <- 1
      keepname <- names(keep)
      if (is.null(keepname)) keepname <- "keep"
      if (length(keep) != n)
        stop("argument \"keep\" has incorrect dimension")
    }
  }
  else {
    nkeep <- ncol(keep)
    keepname <- names(keep)
    if (nrow(keep) != n)
      stop("argument \"keep\" has incorrect dimension")
    if (nkeep == 1)
      keep <- keep[, 1]
  }
  if (is.null(keepname))
    keepname <- paste("keep", as.character(1:nkeep),
                      sep = "")
  if (nkeep > 0) {
    if (is.factor(msres[, 1]))
```

```
      msres[, 1] <- factor(msres[, 1])
     tbl <- table(msres[, 1])
     if (nkeep > 1)
      keep <- keep[ord1,,drop=FALSE]
     if (nkeep == 1) {
      ddcovs <- rep(keep, tbl)
      ddcovs <- ddcovs[ord]
      ddcovs <- as.data.frame(ddcovs)
      names(ddcovs) <- keepname
     }
     else {
      ddcovs <- lapply(1:nkeep, function(i) rep(keep[,
                                          i], tbl))
      ddcovs <- as.data.frame(ddcovs)
      names(ddcovs) <- keepname
     }
     msres <- cbind(msres, ddcovs)
    }
  }
  attr(msres, "trans") <- trans
  class(msres) <- c("msdata","data.frame")
  return(msres)
}

msprepEngine <- function(time,status,id,starttime,startstate,trans,originalStates,longmat)
{
  ### Recursive engine for msprep
  ### Input:
  ###       time: n x K numeric matrx containing arrival times
  ###       status: n x K numeric matrix containing arrival times
  ###       id: n vector with ids
  ###       starttime: n vector of starting times
  ###       startstate: n vector of starting states
  ###       trans: current (K x K) matrix
  ###       originalStates: the numbers of the original states in the
  ###         current transition matrix
  ###       longmat: the matrix in longformat that has already been
  ###         constructed
  ### Output:
  ###       A new longmat matrix with new data appended
  if (is.null(nrow(time))) return(longmat) # finished
  if (nrow(time)==0) return(longmat) # also finished
  states.to <- apply(!is.na(trans),1,sum)
  absorbing <- which(states.to==0)
  states.from <- apply(!is.na(trans),2,sum)
  startings <- which(states.from==0)
  # preset values for next call to msprepEngine
  newstate <- startstate
```

```
  newtime <- starttime
  to.remove <- NULL # indices in data, state, time to be removed at the end
  for (starting in startings) {
    # select all subjects starting in starting, no is nstart
    subjs <- which(startstate==starting)
    nstart <- length(subjs)
    # determine states that can be reached from starting
    tostates <- which(!is.na(trans[starting,]))
    transs <- trans[starting,tostates]
    nreach <- length(tostates)
    # make matrix with nstart*nreach rows
    if ((nstart>0) & (nreach>0)) {
      Tstart <- starttime[subjs]
      Tstop <- time[subjs,tostates,drop=FALSE]
      # event times before start time do not count
      # the statement below defining hlp makes sure
      # these are considered as censorings
      Tstop[Tstop<Tstart] <- Inf
      stat <- status[subjs,tostates,drop=FALSE]
      smallesttime <- apply(Tstop,1,min) # determine first event time
      # now determine which is the corresponding state
      # bit tricky because of censoring and possible data errors
      # limited warnings given at present ...
      hlp <- Tstop * 1/stat
      hlp[Tstop==0 & stat==0] <- Inf # define 0/0 as infinity
      nexttime <- apply(hlp,1,min) # determine first event time
      censored <- which(is.infinite(apply(hlp,1,min)))
      wh <- which(smallesttime<nexttime)
      whminc <- setdiff(wh,censored)
      if (length(whminc)>0) {
        whsubjs <- id[subjs[whminc]]; whsubjs <- paste(whsubjs,collapse=" ")
        warning("From starting state ",originalStates[starting],", subject ",
                whsubjs," has smallest transition time with status=0, larger transition time with
status=1")
      }
      nexttime[censored] <- smallesttime[censored]
      if (ncol(hlp)>1) {
        hlpsrt <- t(apply(hlp,1,sort))
        warn1 <- which(hlpsrt[,1]-hlpsrt[,2]==0)
        if (length(warn1)>0)
        {
          isw <- id[subjs[warn1]]; isw <- paste(isw,collapse=" ")
          hsw <- hlpsrt[warn1,1]; hsw <- paste(hsw,collapse=" ")
          warning("Starting from state ",originalStates[starting],
                  ", simultaneous transitions possible for subjects ",
                  isw," at times ",hsw,
                  "; smallest receiving state chosen")
        }
```

```
      }
      if (length(censored)>0) {
        nextstate <- apply(hlp[-censored,,drop=FALSE],1,which.min)
        reachAbsorb <- (1:nstart)[-censored][which(tostates[nextstate] %in% absorbing)]
      } else {
        nextstate <- apply(hlp,1,which.min)
        reachAbsorb <- (1:nstart)[which(tostates[nextstate] %in% absorbing)]
      }
      # the status to be returned in long dataframe has 0 if censored
      # and 1 for the transition followed
      statmat <- matrix(0,nstart,nreach)
      if (length(censored)>0) statmatmin <- statmat[-censored,,drop=FALSE] else statmatmin <-
statmat
      if (nrow(statmatmin)>0)
        statmatmin <- t(sapply(1:nrow(statmatmin),function(i) {
          x <- statmatmin[i,]
          x[nextstate[i]] <- 1
          return(x) }
        ))
      if (length(censored)>0) statmat[-censored,] <- statmatmin else statmat <- statmatmin
      mm <- matrix(c(
        rep(id[subjs],rep(nreach,nstart)), # id
        rep(originalStates[starting],nreach*nstart), # from
        rep(originalStates[tostates],nstart), # to
        rep(transs,nstart), # trans
        rep(Tstart,rep(nreach,nstart)), # Tstart
        rep(nexttime,rep(nreach,nstart)), # Tstop
        as.vector(t(statmat))), # status
        nreach*nstart,7)
      # stack upon what is already there
      longmat <- rbind(longmat,mm)
      # adjust data: remove subjects who didn't reach any new state
      #             for those who do reach new state, adjust starting
      #             state and time
      to.remove <- c(to.remove,subjs[c(censored,reachAbsorb)])
      if (length(censored)>0) newstate[subjs[-censored]] <- tostates[nextstate]
      else newstate[subjs] <- tostates[nextstate]
      if (length(censored)>0) newtime[subjs[-censored]] <- nexttime[-censored]
      else newtime[subjs] <- nexttime
    }
  }
  if (length(to.remove)>0) {
    time <- matrix(time[-to.remove,], ncol = dim(time)[2])
    status <- matrix(status[-to.remove,], ncol= dim(time)[2])
    newtime <- newtime[-to.remove]
    newstate <- newstate[-to.remove]
    id <- id[-to.remove]
  }
```

```
    # Some states will be removed from the transition matrix in the next call
    # We have to adjust newstate to mean the new states
    K <- nrow(trans)
    idx <- rep(1,K); idx[startings] <- 0; idx <- cumsum(idx)
    newstate <- idx[newstate]
    Recall(time=time[,-startings],status=status[,-startings],
           id=id,starttime=newtime,startstate=newstate,
           trans=trans[-startings,-startings],
           originalStates=originalStates[-startings],
           longmat=longmat)
  }

}

library(survival)
setwd("Enter working directory here")

### Some values for testruns #####
##

n. = 4000000
# from lambda = 0.0000005(8%) to 0.0000028(12%), 0.00000128 hits around 10%
lambda_AD. =  0.00000128

lambda_CTCL. = 3/1000000

propDiag. = 2/8
propTRTChange. = 0.1
propTCS. = 19/20
propCTacro. = 0.30
propChoose. = 0.4
yearsObs. = 12 # if not NULL then the function will left-truncate at random starting points and
right-censor after specified years.

n = n.; lambda_AD = lambda_AD.; lambda_CTCL = lambda_CTCL.;
propDiag = propDiag.; propTRTChange = propTRTChange.; propTCS = propTCS.;
yearsObs = yearsObs.;propCTacro = propCTacro.;propChoose = propChoose.



### Simulation function ####

simulation2 = function(seed = 11112019, n = n., lambda_AD = lambda_AD., lambda_CTCL =
lambda_CTCL.,
                      propDiag = propDiag., propTRTChange = propTRTChange., propTCS = propTCS.,
propCTacro = propCTacro.,
                      propChoose = propChoose., yearsObs = yearsObs., scale_S_2 = 8){
  set.seed(seed)
```

```
## function time under here.
print("Simulating data")
# proportion of miss-diagnosis of CTCL
Q = rbinom(n = n, size = 1, prob = propDiag)

# proportion of treatment changes, i.e. subjects not shifting due to response to treatment of TCS
P = rbinom(n = n, size = 1, prob = propTRTChange)

#proportion of TCS as first line of treatment when diagnosed with AD.
S = rbinom(n = n, size = 1, prob = propTCS)

#proportion of CTCL-misdiagnosed whom could be tacro treated.
R = rbinom(n, 1, propCTacro)

if (!is.null(yearsObs)){
  timeStart = runif(n, max = 100)
  timeStop = timeStart + yearsObs
}

######################## Fourth approach ###########################
## Same as the first just with the addition of a direct state from healthy to TRT (Tacro)
## with the addition of left and right truncation in the observation period (set to 12 years most likely)
############ Times ##############

# time to AD
u_AD = runif(n = n)
T_AD = (-log(u_AD))^4/(lambda_AD) # -log(u_AD)/(lambda_AD)

#time to censoring
T_CENS = (1 - exp(log(1-runif(n))*2))*100

#Time to CTCL

u_CTCL = runif(n = n)

C1 = -log(u_AD)/lambda_CTCL

T_CTCL = rep(NA, n)

T_CTCL = -log(u_CTCL)/lambda_CTCL

#time to treatment for AD
T_AD_OBS =  T_AD * as.numeric(T_AD<T_CTCL) +
  Q*T_AD*as.numeric(T_CTCL<=T_AD) +      # CTCL treated proportion of CTCL patients.
  (1-Q)*T_CTCL*as.numeric(T_CTCL<=T_AD)  # AD treated proportion of CTCL patients.
```

```
  T_TCS = T_AD_OBS*S + T_CENS*(1-S)

  T_TACRO = T_AD_OBS*(1-S) + T_CENS*S

  #Time to non-response for first line treatment for AD
  choose = rbinom(n, 1, propChoose)
  S_1 = (1 - exp(log(1-runif(n))*2))*(1-choose) + (rexp(n, 3) + 1)*choose

  #Time to non-response for second line treatment for AD

  maxwei = function(a= 1, b = 10, shape = 1,scale=1){
    replicate(a, max(rweibull(b, shape, scale)))
  }

  S_2 = maxwei(n, 2, 0.9, scale_S_2)

  #Time to change of treatment for AD, first line of treatment
  T_TRT = pmax(((((T_TCS + S_1)*P +
                 T_CENS*(1-P))*as.numeric(T_TCS==T_AD) +
                (T_TCS + S_1)*as.numeric(T_TCS==T_CTCL)*R +
                (T_CENS)*as.numeric(T_TCS==T_CTCL)*(1-R))*S,
              T_TACRO*(1-S))

  #Time to change of treatment for AD or new diagnosis
  T_STOP =  T_CENS*as.numeric(T_AD==T_AD_OBS) +
    (T_AD_OBS + S_2)*as.numeric(T_AD_OBS==T_CTCL)

  #Time to observed CTCL
  T_CTCL_OBS = T_CTCL*as.numeric(T_AD_OBS==T_AD) +
T_STOP*as.numeric(T_AD_OBS==T_CTCL)

  # grid.arrange(qplot(T_AD, xlim = c(0,100)), qplot(T_CTCL, xlim = c(0,100)), qplot(T_CENS),
  #             qplot(T_AD_OBS, xlim = c(0,100)), qplot(T_STOP, xlim = c(0,100)),
qplot(T_CTCL_OBS, xlim = c(0,100)),
  #             qplot(T_TRT, xlim = c(0,100)),
  #             qplot(S_1), qplot(S_2, binwidth = 0.1))

  ########## preparing data ###########
  print("msprep step")
  simpDat <- data.frame(TCS_t=T_TCS,
                        TCS_s=as.numeric(T_TCS < T_CENS),
                        CTCL_t=T_CTCL_OBS,
                        CTCL_s=as.numeric(T_CTCL_OBS < T_CENS),
                        TRT_t = T_TRT,
                        TRT_s = as.numeric(T_TRT < T_CENS)
  )
```

```
  simpDat$CTCL_t = T_CTCL_OBS * as.numeric(T_CTCL_OBS<=T_CENS) + T_CENS *
as.numeric(T_CENS<T_CTCL_OBS)

  simpDat$TCS_t = T_TCS * as.numeric(T_TCS<T_CENS) + T_CENS *
as.numeric(T_CENS<=T_TCS)

  simpDat$TRT_t = T_TRT * as.numeric(T_TRT<T_CENS) + T_CENS *
as.numeric(T_CENS<=T_TRT)

  if (!is.null(yearsObs)){
    simpDat = cbind(simpDat, timeStart, timeStop)
  }

  ########## Preparing mstates ################

  simp = transMat(x = list(c(2,3, 4), c(3, 4), c(4), c()),
                names = c("Healthy", "TCS", "Tacro", "CTCL"))

  print("msprep call")
  if (!is.null(yearsObs)){
    simpPrep = msprep(time=c(NA, "TCS_t", "TRT_t", "CTCL_t"),
                      status=c(NA, "TCS_s", "TRT_s", "CTCL_s"),
                      data = simpDat, trans=simp, keep = c("timeStart", "timeStop"))
  } else {
    simpPrep = msprep(time=c(NA, "TCS_t", "TRT_t", "CTCL_t"),
                      status=c(NA, "TCS_s", "TRT_s", "CTCL_s"),
                      data = simpDat, trans=simp)
  }


  ####### indicators ####

  print("Data prep")
  statusdat = reshape(data = simpPrep[,c("status", "id", "from", "trans")], v.names = "status",
                      idvar = c("id", "from"), timevar = "trans", direction = "wide")
  statusdat = statusdat[, c("id","from", "status.1", "status.2", "status.3", "status.4", "status.5",
"status.6")]

  fromdat = as.data.frame(cbind(id = simpPrep[simpPrep$from==2 & simpPrep$to == 3 &
simpPrep$status == 1, c("id")],
                                prior = 1, from = 3))

  simpPrep = merge(x = simpPrep, y = statusdat, by = c("id", "from"), sort = T)
  simpPrep = simpPrep[order(simpPrep$id, simpPrep$from, simpPrep$to),]

  colnames(simpPrep)[colnames(simpPrep) %in% c("status.1", "status.2", "status.3", "status.4",
"status.5", "status.6")] =
    c("1", "2", "3", "4", "5", "6")
```

```
  simpPrep$trans1[simpPrep$from==1 & (simpPrep$to==3 & simpPrep$status == 1) |
(simpPrep$to==2 & simpPrep$status == 1)] = 1
  simpPrep$trans1[simpPrep$from==1 & simpPrep$to==4 & (simpPrep$status == 1 |
                                        simpPrep$`1` == 0 & simpPrep$`2` == 0 & simpPrep$`3` ==
0)] = 2
  simpPrep$trans1[simpPrep$from==3 & simpPrep$to==4 |
              (simpPrep$from == 2 & simpPrep$to == 4 & simpPrep$`4`== 0) |
              simpPrep$from == 2 & simpPrep$to == 3 & simpPrep$`4` == 1] = 3

  simpPrep$statusNew = ifelse(simpPrep$trans1 == 1 & (simpPrep$`1`==1 | simpPrep$`2`==1) |
                       simpPrep$trans1 == 2  & (simpPrep$`3`==1 ) |
                       simpPrep$trans1 == 3 & (simpPrep$`4`==1 |simpPrep$`5`==1 |
simpPrep$`6`==1 ), 1, NA
  )

  simpPrep$statusNew = ifelse(is.na(simpPrep$statusNew) & !is.na(simpPrep$trans1), 0,
simpPrep$statusNew)

  simpPrep = merge(x = simpPrep, y = fromdat, by = c("id", "from"), all.x = T)

  simpPrep$prior = ifelse(is.na(simpPrep$prior), 0, simpPrep$prior)

  simpPrep$stat = as.numeric(simpPrep$to==4 & simpPrep$statusNew == 1)

  simpPrep$trt1 = as.factor(ifelse(simpPrep$from==3, 1, ifelse(simpPrep$from==2 &
simpPrep$from!=3, 0, NA)))

  simpPrep = simpPrep[, !(colnames(simpPrep) %in% c("1","2","3","4","5","6"))]

  if (!is.null(yearsObs)){

    cutsimpPrep = subset(simpPrep, Tstart <= timeStart & Tstop >= timeStart & Tstop <= timeStop |
                  Tstart >= timeStart & Tstart <= timeStop & Tstop >= timeStop |
                  Tstart <= timeStart & Tstop >= timeStop |
                  Tstart >= timeStart & Tstop <= timeStop)

    cutsimpPrep$cutTstart = with(cutsimpPrep, timeStart*as.numeric(timeStart > Tstart) +
                        Tstart*as.numeric(timeStart <= Tstart))

    cutsimpPrep$cutTstop = with(cutsimpPrep, timeStop*as.numeric(timeStop < Tstop) +
                        Tstop*as.numeric(timeStop >= Tstop))

    cutsimpPrep$cutStatus = with(cutsimpPrep, as.numeric(status == 1 & Tstop <= timeStop))

    cutsimpPrep$cutStat = ifelse(cutsimpPrep$stat == 1 &
cutsimpPrep$Tstop==cutsimpPrep$cutTstop,1,
                        ifelse(is.na(cutsimpPrep$stat), NA, 0))
```

```
  cutsimpPrep$Tstart = cutsimpPrep$cutTstart
  cutsimpPrep$Tstop = cutsimpPrep$cutTstop
  cutsimpPrep$status = cutsimpPrep$cutStatus
  cutsimpPrep$stat = cutsimpPrep$cutStat

  cutsimpPrep$time = cutsimpPrep$cutTstop - cutsimpPrep$cutTstart

  cutsimpPrep$QTstart = cutsimpPrep$cutTstart - cutsimpPrep$timeStart
  cutsimpPrep$QTstop = cutsimpPrep$cutTstop - cutsimpPrep$timeStart

  cutsimpPrep = cutsimpPrep[,colnames(cutsimpPrep)[!(colnames(cutsimpPrep) %in%
                                            c("timeStart", "timeStop", "cutTstart",
                                              "cutTstop", "cutStatus", "cutStat"))]]

  cutsimpPrep = cutsimpPrep[cutsimpPrep$time > 1*10^{-3}, ]

  cutsimpPrep$from = as.factor(cutsimpPrep$from)

  cutsimpPrep$to = as.factor(cutsimpPrep$to)

  cutsimpPrep$trt1 = factor(cutsimpPrep$trt1)

  cutsimpPrep$trans = as.factor(cutsimpPrep$trans)

  cutsimpPrep$trans1 = as.factor(cutsimpPrep$trans1)
 }

 simpPrep = simpPrep[simpPrep$time > 1*10^{-3},]

 simpPrep$from = as.factor(simpPrep$from)

 simpPrep$to = as.factor(simpPrep$to)

 simpPrep$trt1 = factor(simpPrep$trt1)

 simpPrep$trans = as.factor(simpPrep$trans)

 simpPrep$trans1 = as.factor(simpPrep$trans1)

 ################ Analysis ###################

 print("Analysis")
 simpCox1 = summary(coxph(Surv(Tstart, Tstop, stat) ~ trt1,
                data = subset(simpPrep, trans1 == 3), method = "breslow"))[c(7,8)]

 simpCox1vec = cbind(trt= "TACRO", as.data.frame(matrix(simpCox1$conf.int[1, c(1,3,4)], ncol =
3)))
```

```
  colnames(simpCox1vec) = c("trt", "est_1", "l.95_1", "u.95_1")
  simpCox1vec


  if (!is.null(yearsObs)){
    print("Time-interval analysis")

    simpCox2.4 = summary(coxph(Surv(QTstart, QTstop, stat) ~ trt1,
                         data = subset(cutsimpPrep, trans1 == 3), control = coxph.control(timefix =
FALSE)))
    simpCox2.4vec = cbind(trt= c("Tacro"), as.data.frame(matrix(simpCox2.4$conf.int[c(1),
c(1,3,4)], ncol = 3)))

    colnames(simpCox2.4vec) = c("trt", "est_2", "l.95_2", "u.95_2")

    simpCox2.4vec

  }

  sum1 = sum(subset(simpPrep, simpPrep$trans1=="3" & simpPrep$trt1=="1")[,"time"])
  sum1_r = sum(subset(simpPrep, simpPrep$trans1=="3" & simpPrep$trt1=="1")[,"stat"])
  rateTacro = sum1_r/sum1

  sum2 = sum(subset(simpPrep, simpPrep$trans1=="3" & simpPrep$trt1=="0")[,"time"])
  sum2_r = sum(subset(simpPrep, simpPrep$trans1=="3" & simpPrep$trt1=="0")[,"stat"])
  rateTCS = sum2_r/sum2

  sum3 = sum(subset(cutsimpPrep, cutsimpPrep$trans1=="3" & cutsimpPrep$trt1=="1")[,"time"])
  sum3_r = sum(subset(cutsimpPrep, cutsimpPrep$trans1=="3" & cutsimpPrep$trt1=="1")[,"stat"])
  rateTacroC = sum3_r/sum3

  sum4 = sum(subset(cutsimpPrep, cutsimpPrep$trans1=="3" & cutsimpPrep$trt1=="0")[,"time"])
  sum4_r = sum(subset(cutsimpPrep, cutsimpPrep$trans1=="3" & cutsimpPrep$trt1=="0")[,"stat"])
  rateTCSC = sum4_r/sum4


  print("Returning")
  if (!is.null(yearsObs)){
    return(cbind(seed = seed, n = n, lambda_AD = lambda_AD, lambda_CTCL = lambda_CTCL,
                 propDiag = propDiag, propTRTChange = propTRTChange,
                 propTCS = propTCS, propCTacro = propCTacro, propChoose = propChoose.,
                 yearsObs = yearsObs, scale_S_2 = scale_S_2, simpCox1vec[,2:4],
simpCox2.4vec[,2:4],
                 rateTacro = rateTacro, rateTCS = rateTCS, rateTacroC = rateTacroC, rateTCSC =
rateTCSC)
    )
  } else {
    return(cbind(rbind(cbind(mod = "mod1.1", simpCox1vec)),
```

```
    variables = cbind(n = n, lambda_AD = lambda_AD, lambda_CTCL = lambda_CTCL,
                  propDiag = propDiag, propTRTChange = propTRTChange,
                  propTCS = propTCS, propCTacro = propCTacro, propChoose = propChoose.,
                  yearsObs = yearsObs)))
  }
}


### run ##########

nruns = 1000

simDat = data.frame(propTCS. = c(rep(0.95,7), 0.99,0.9, rep(0.9,7), 0.99, 0.95, rep(0.95, 2)),
                propTRTChange. = c(rep(0.05,3), 0.1,0.2, rep(0.05,4), rep(0.2,3), 0.05,0.1, rep(0.2,4),
rep(0.05, 2)),
                propDiag. = c(0.25,0.5,0.75, rep(0.25,7), 0.5, 0.75, rep(0.25,6), rep(0.25, 2)),
                propCTacro. = c(rep(0.25,5), 0.1, 0.4, rep(0.25,2), rep(0.4,5), 0.1, 0.25, rep(0.4,2),
rep(0.25, 2)),
                scale_S_2. = c(rep(3.5,18), 1.2, 0.5)
                )

simDat = cbind(simDat, lambda_AD. =  0.00000128, lambda_CTCL. = 6/1000000, propChoose. =
0.4, yearsObs. = 12, n. = 4000000)

simThis = data.frame(NA)

set.seed(11112019)
for (i in 1:dim(simDat)[1]){
  simThis[(1 + (nruns)*(i-1)):((nruns)*(i)),1:length(colnames(simDat))] = simDat[rep(i, nruns), ]
}

colnames(simThis) = colnames(simDat)

simThis = cbind(seed. = sample(1:1000000000, dim(simThis[1])), simThis)

to= dim(simThis)[1]
to

write.csv(simThis, file = "simThisTest2.csv")

for (i in 1:to){
  print(i)

  tmp = simulation2(seed = simThis[i,"seed."], n = simThis[i,"n."], lambda_AD =
simThis[i,"lambda_AD."], lambda_CTCL = simThis[i,"lambda_CTCL."],
                 propDiag = simThis[i,"propDiag."], propTRTChange =
simThis[i,"propTRTChange."],
                 propTCS = simThis[i,"propTCS."], propCTacro = simThis[i,"propCTacro."],
```

```
                        propChoose = simThis[i,"propChoose."], yearsObs = simThis[i,"yearsObs."],
scale_S_2=simThis[i,"scale_S_2."])

  if(i == 1){
    simData = data.frame(matrix(NA, ncol = length(colnames(tmp))))
    colnames(simData) = colnames(tmp)
  }

  simData[i,] = tmp

  save(simData, file = "Simulation1_3.rData")
}

write.csv(simData, file = "simDataTest2.csv")
```
