# Supplementary material for: Reverse causation bias: A simulation study comparing first- and second-line treatments with an overlap of symptoms between treatment indication and studied outcome
Source: PLoS One. 2024 Jul 12;19(7):e0304145. doi: 10.1371/journal.pone.0304145 (PMC11244844; doi:10.1371/journal.pone.0304145)
Supplement: Attachment — Submitted filename: Response to Reviewers.docx [file pone.0304145.s006.docx]

Response to Reviewers

Dear Reviewers and Editors,

Thank you very much for reviewing and commenting on our manuscript. Please find our answers to your comments below.

Kind regards,

Christian Øland, on behalf of the authors

Responses to reviewer, where applicable, have been written in blue with “Answer:” in front.

# 1 Points Raised by Academic Editor

Journal requirements:

When submitting your revision, we need you to address these additional requirements.

1. Please ensure that your manuscript meets PLOS ONE's style requirements, including those for file naming. The PLOS ONE style templates can be found at

<https://journals.plos.org/plosone/s/file?id=wjVg/PLOSOne_formatting_sample_main_body.pdf> and

<https://journals.plos.org/plosone/s/file?id=ba62/PLOSOne_formatting_sample_title_authors_affiliations.pdf>

Answer: We have reviewed the style requirements and made edits to the manuscript accordingly.

2. Please note that PLOS ONE has specific guidelines on code sharing for submissions in which author-generated code underpins the findings in the manuscript. In these cases, all author-generated code must be made available without restrictions upon publication of the work. Please review our guidelines at <https://journals.plos.org/plosone/s/materials-and-software-sharing#loc-sharing-code> and ensure that your code is shared in a way that follows best practice and facilitates reproducibility and reuse.

Answer: Thank you. Please note that the code used in the simulations has been shared in its entirety in the supplement under S1_file (previously S5_file).

3. We note that the grant information you provided in the ‘Funding Information’ and ‘Financial Disclosure’ sections do not match.

When you resubmit, please ensure that you provide the correct grant numbers for the awards you received for your study in the ‘Funding Information’ section.

Answer: Thank you. We have ensured the ‘Funding Information' is correct.

4. Thank you for stating the following in the Competing Interests section:

“All authors are or have been employees of LEO Pharma A/S and may be shareholders of LEO Pharma A/S. LEO Pharma A/S owns and sells topical tacrolimus/Protopic

®.”

Please confirm that this does not alter your adherence to all PLOS ONE policies on sharing data and materials, by including the following statement: "This does not alter our adherence to  PLOS ONE policies on sharing data and materials.” (as detailed online in our guide for authors <http://journals.plos.org/plosone/s/competing-interests>).  If there are restrictions on sharing of data and/or materials, please state these. Please note that we cannot proceed with consideration of your article until this information has been declared.

Please include your updated Competing Interests statement in your cover letter; we will change the online submission form on your behalf.

Answer: We have added the requested statement and updated our competing interests statement. We thank you for helping in ensuring transparency in our work which we wholeheartedly support.

5. We noted in your submission details that a portion of your manuscript may have been presented or published elsewhere. [Abstract has been presented at ICPE 2023.] Please clarify whether this publication was peer-reviewed and formally published. If this work was previously peer-reviewed and published, in the cover letter please provide the reason that this work does not constitute dual publication and should be included in the current manuscript.

Answer: The abstract was presented as a poster at a conference. It does not constitute dual publication. We have updated the cover letter to be clearer on this matter.

6. For studies involving third-party data, we encourage authors to share any data specific to their analyses that they can legally distribute. PLOS recognizes, however, that authors may be using third-party data they do not have the rights to share. When third-party data cannot be publicly shared, authors must provide all information necessary for interested researchers to apply to gain access to the data. (<https://journals.plos.org/plosone/s/data-availability#loc-acceptable-data-access-restrictions>)

For any third-party data that the authors cannot legally distribute, they should include the following information in their Data Availability Statement upon submission:

a) A description of the data set and the third-party source

b) If applicable, verification of permission to use the data set

c) Confirmation of whether the authors received any special privileges in accessing the data that other researchers would not have

d) All necessary contact information others would need to apply to gain access to the data

Answer: We do not have any third-party data which we cannot legally distribute.

7. Please include captions for your Supporting Information files at the end of your manuscript, and update any in-text citations to match accordingly. Please see our Supporting Information guidelines for more information: <http://journals.plos.org/plosone/s/supporting-information>.

Answer: We have updated accordingly both naming and reference to the supporting information in the manuscript and submission. Thanks.

8. Please review your reference list to ensure that it is complete and correct. If you have cited papers that have been retracted, please include the rationale for doing so in the manuscript text, or remove these references and replace them with relevant current references. Any changes to the reference list should be mentioned in the rebuttal letter that accompanies your revised manuscript. If you need to cite a retracted article, indicate the article’s retracted status in the References list and also include a citation and full reference for the retraction notice.

Answer: The reference list is complete and correct.

# 2 Points Raised by Reviewers

[Note: HTML markup is below. Please do not edit.]

Reviewers' comments:

Reviewer's Responses to Questions

**Comments to the Author**

1. Is the manuscript technically sound, and do the data support the conclusions?

The manuscript must describe a technically sound piece of scientific research with data that supports the conclusions. Experiments must have been conducted rigorously, with appropriate controls, replication, and sample sizes. The conclusions must be drawn appropriately based on the data presented.

Reviewer #1: Yes

Reviewer #2: Yes

2. Has the statistical analysis been performed appropriately and rigorously?

Reviewer #1: Yes

Reviewer #2: Yes

3. Have the authors made all data underlying the findings in their manuscript fully available?

The [PLOS Data policy](https://eur03.safelinks.protection.outlook.com/?url=http%3A%2F%2Fwww.plosone.org%2Fstatic%2Fpolicies.action%23sharing&data=05%7C02%7Cqbodk%40leo-pharma.com%7Cb62e21ccdcf240cc0baa08dc5362d521%7Cd78f7362832c47158e12cc7bd574144c%7C0%7C0%7C638476931480650241%7CUnknown%7CTWFpbGZsb3d8eyJWIjoiMC4wLjAwMDAiLCJQIjoiV2luMzIiLCJBTiI6Ik1haWwiLCJXVCI6Mn0%3D%7C0%7C%7C%7C&sdata=%2FKoci4igx2h6lnMh2NdgJDWoh6rnY%2FpImxmgwVSNDx0%3D&reserved=0) requires authors to make all data underlying the findings described in their manuscript fully available without restriction, with rare exception (please refer to the Data Availability Statement in the manuscript PDF file). The data should be provided as part of the manuscript or its supporting information, or deposited to a public repository. For example, in addition to summary statistics, the data points behind means, medians and variance measures should be available. If there are restrictions on publicly sharing data—e.g. participant privacy or use of data from a third party—those must be specified.

Reviewer #1: Yes

Reviewer #2: Yes

4. Is the manuscript presented in an intelligible fashion and written in standard English?

PLOS ONE does not copyedit accepted manuscripts, so the language in submitted articles must be clear, correct, and unambiguous. Any typographical or grammatical errors should be corrected at revision, so please note any specific errors here.

Reviewer #1: Yes

Reviewer #2: Yes

5. Review Comments to the Author

Please use the space provided to explain your answers to the questions above. You may also include additional comments for the author, including concerns about dual publication, research ethics, or publication ethics. (Please upload your review as an attachment if it exceeds 20,000 characters)

Reviewer #1: This manuscript is very well written and describes a fascinating modeling exercise that addresses an important clinical issue. I appreciated the opportunity to read this excellent work. The findings of this manuscript are important for treating atopic dermatitis and are relevant much more generally.

Answer: Thank you very much.

Reviewer #2: This study investigated the impact of reverse causation on the association between the use of topical tacrolimus and CTCL in a multinational, population-based study using TCS as comparator.

Fig 1 is not correct and the authors should correct it so that it is clearly visible.

Answer: We have updated the title and the mentioning of the figure in the manuscript.

6. PLOS authors have the option to publish the peer review history of their article ([what does this mean?](https://eur03.safelinks.protection.outlook.com/?url=https%3A%2F%2Fjournals.plos.org%2Fplosone%2Fs%2Feditorial-and-peer-review-process%23loc-peer-review-history&data=05%7C02%7Cqbodk%40leo-pharma.com%7Cb62e21ccdcf240cc0baa08dc5362d521%7Cd78f7362832c47158e12cc7bd574144c%7C0%7C0%7C638476931480658465%7CUnknown%7CTWFpbGZsb3d8eyJWIjoiMC4wLjAwMDAiLCJQIjoiV2luMzIiLCJBTiI6Ik1haWwiLCJXVCI6Mn0%3D%7C0%7C%7C%7C&sdata=vF5w6jekctpU2Pw5Nmd4LQdgzl9P6W24wZ8XDnTp%2BRk%3D&reserved=0)). If published, this will include your full peer review and any attached files.


If you choose “no”, your identity will remain anonymous but your review may still be made public.


**Do you want your identity to be public for this peer review?** For information about this choice, including consent withdrawal, please see our [Privacy Policy](https://eur03.safelinks.protection.outlook.com/?url=https%3A%2F%2Fwww.plos.org%2Fprivacy-policy&data=05%7C02%7Cqbodk%40leo-pharma.com%7Cb62e21ccdcf240cc0baa08dc5362d521%7Cd78f7362832c47158e12cc7bd574144c%7C0%7C0%7C638476931480672168%7CUnknown%7CTWFpbGZsb3d8eyJWIjoiMC4wLjAwMDAiLCJQIjoiV2luMzIiLCJBTiI6Ik1haWwiLCJXVCI6Mn0%3D%7C0%7C%7C%7C&sdata=ngcuR9wRAjbQrpAT7k2p7wQkgDjpSuCJUT4tbNb2sQ0%3D&reserved=0).

Reviewer #1: **Yes:** Steven Feldman

Reviewer #2: No
